# Associations Between Diverse Beverage Consumption Patterns and Oral Health: Evidence from a National Survey in Hungary

**DOI:** 10.3390/nu17152572

**Published:** 2025-08-07

**Authors:** Amr Sayed Ghanem, Zsuzsa Emma Hajzer, Vanessza Hadar, Eszter Vargáné Faludi, Tamari Shenheliia, Marianna Móré, Attila Csaba Nagy, Ágnes Tóth

**Affiliations:** 1 Department of Epidemiology, Faculty of Health Sciences, University of Debrecen, 4028 Debrecen, Hungary; aghanem@etk.unideb.hu (A.S.G.); vanesszahadar16@gmail.com (V.H.); faludi.eszter@etk.unideb.hu (E.V.F.); tam.shenheliia@gmail.com (T.S.); nagy.attila@etk.unideb.hu (A.C.N.); 2 Department of Dietetics, Faculty of Health Sciences, University of Debrecen, 4028 Debrecen, Hungary; hajzer.zsuzsa@etk.unideb.hu; 3 Department of Gerontology, Faculty of Health Sciences, University of Debrecen, 4400 Nyíregyháza, Hungary; more.mariann@etk.unideb.hu; 4 Department of Nursing and Integrative Health Sciences, Faculty of Health Sciences, University of Debrecen, 4028 Debrecen, Hungary

**Keywords:** oral health, beverage consumption, daily intake, sugar-sweetened beverages, dental caries, gum bleeding, periodontitis, smoking, alcohol use, population health

## Abstract

**Background/Objectives**: Oral diseases are highly prevalent in Hungary and driven in part by unhealthy beverage consumption, smoking, and other behaviors. No prior study has examined the impact of beverage consumption patterns on oral health in a representative Hungarian population. This study investigated the association between beverage intake, lifestyle factors, and oral health outcomes among Hungarian adults. **Methods**: Data were drawn from the 2019 Hungarian European Health Interview Survey, a nationally representative cross-sectional study. Oral health outcomes and key exposures, including beverage consumption, smoking, alcohol use, and sociodemographic variables, were self-reported. Associations were assessed using multiple logistic regression models. **Results**: Among 5425 adults, higher dairy intake was linked to less gum bleeding (odds ratio = 0.76; 95% confidence intervals [0.59–0.96]) and lower odds of teeth missing (0.63 [0.47–0.86]). Weekly juice intake reduced gum bleeding (0.62 [0.51–0.76]) and missing teeth (0.83 [0.71–0.96]). Daily soda was associated with more gum bleeding (1.94 [1.53–2.47]), caries (1.57 [1.27–1.94]), and poor self-perceived oral health (1.32 [1.10–1.59]). Alcohol (1–4 times/week) increased gum bleeding (1.38 [1.07–1.77]) and tooth mobility (1.47 [1.02–2.11]). Smoking raised odds for caries (1.42 [1.21–1.66]) and missing teeth (1.81 [1.55–2.10]). **Conclusions**: Increasing dairy and fresh juice intake while reducing sugar-sweetened and acidic beverages, alongside tobacco and alcohol control and routine oral health screening, are effective strategies for improving population oral health across all sociodemographic groups.

## 1. Introduction

Oral diseases remain the most prevalent health conditions worldwide, affecting an estimated 3.5 billion people in 2019, making them more widespread than any of the other 300 plus diseases tracked globally [1]. Untreated caries of permanent teeth is the single most common condition, with around 2 billion affected, while severe periodontitis and untreated caries of deciduous teeth impact approximately 1 billion and 510 million people, respectively. Edentulism (complete tooth loss) affects an additional 350 million people [1,2]. It is worth mentioning that the combined global burden of oral diseases exceeds that of all five major non-communicable diseases—mental disorders, cardiovascular diseases, diabetes, chronic respiratory diseases, and cancers—by approximately one billion cases. This highlights their unparalleled scope and significance [2]. The global average prevalence rates in 2019 were 28.7% for caries of permanent teeth, 18.8% for severe periodontitis, and 6.8% for edentulism, with prevalence remaining high and persistent across all World Bank income groups and WHO regions [2].

While prevalence rates for some conditions, such as caries and edentulism, have remained relatively stable or declined modestly, the absolute number of cases has grown substantially. This growth outpaces demographic expansion, particularly in low- and middle-income countries. From 1990 to 2019, caries cases increased globally by 46%, severe periodontitis cases by over 99%, and edentulism cases by 81%, with these increases largely driven by population growth in lower-resource settings [2]. In Europe, the burden of oral disease remains particularly pronounced, with the highest prevalence of caries of permanent teeth (33.6%, 293.9 million cases), a substantial burden of severe periodontitis (17.9%, 136.2 million cases), and edentulism (12.4%, 88.2 million cases) [2]. Although Europe experienced only a modest 6% increase in caries cases and 30% in edentulism cases between 1990 and 2019, the region continues to carry a significant share of global disease burden.

In Hungary, the burden of oral diseases remains one of the highest in Europe and is reflected in both high prevalence rates and significant economic impact. As of 2019, untreated caries of permanent teeth affected 37.9% of individuals aged 5 years and older, while untreated caries of deciduous teeth was observed in 47.8% of children aged 1–9 years [3,4]. Severe periodontal disease was present in 8.6% of the population aged 15 years and above, defined by clinical parameters such as a gingival pocket depth of at least 6 mm, CPI score of 4, or clinical attachment loss greater than 6 mm [3]. Edentulism, or complete tooth loss, affected 14.1% of adults aged 20 years and older. These figures, derived from the Global Burden of Disease 2021 dataset and based on standardized diagnostic criteria, underscore the widespread nature of oral pathology across age groups in Hungary [4].

The economic impact is also considerable. In 2019, total expenditure on dental healthcare in Hungary reached 364 million USD, corresponding to a per capita spending of 37 USD [3]. Furthermore, productivity losses attributed to the five major oral diseases were estimated at 720 million USD, highlighting the broader societal cost beyond direct healthcare expenses. These estimates are calculated by integrating disease prevalence data from the IHME GBD study [4] with UN DESA population projections. They encompass direct clinical costs as well as indirect economic burdens from work absenteeism and reduced productivity.

### Commercial and Behavioral Risk Factors for Oral Diseases in Hungary

A range of commercial and behavioral factors contribute significantly to the burden of oral diseases in Hungary, reflecting both global and country-specific trends. High sugar consumption, which is driven largely by the widespread availability and aggressive marketing of sugar-sweetened beverages, remains a major risk factor, with Hungary reporting the highest per capita soda consumption worldwide (310 L per person per year, with 12.8% of the population consuming soda daily) [5,6]. Dairy intake is moderate, with an average annual per capita consumption of 172 kg of milk [7]. Alcohol consumption in Hungary is close to the European average, with an annual per capita intake of 5.04 L, but with marked sex differences (men: 8.23 L/year, women: 2.09 L/year) [8]. Tobacco use continues to be a major public health issue, exacerbating oral disease risk, especially in socially disadvantaged groups [9]. Energy drink consumption presents a unique and growing challenge in Hungary, with the country ranking among the highest in Europe for both youth and adult consumption [10]. Recent years have seen legislative efforts to restrict sales of energy drinks to minors, prompted by alarming statistics: in 2018, 78% of Hungarian adolescents reported consuming energy drinks, and 13% were classified as high-frequency consumers [10]. The annual national energy drink market exceeds 64 million liters, and regular overconsumption has led to hundreds of pediatric emergency visits [10]. The interplay of these commercial determinants, alongside individual behaviors, creates an environment where exposure to cariogenic, erosive, and potentially harmful substances is nearly ubiquitous. These factors are compounded by the marketing practices of multinational food and beverage companies, which target vulnerable populations through tailored promotions and sponsorships. Hungary’s response, including taxation and public health campaigns, has achieved only limited success to date, as evidenced by persistently high disease prevalence and consumption rates.

Despite the high prevalence of oral diseases and the recognition of key behavioral risk factors, no previous study from Hungary has systematically investigated the association between beverage consumption patterns and oral health outcomes in a nationally representative adult population. Existing research has predominantly focused on individual risk factors or limited oral health indicators and often relied on non-representative samples or clinical data. Comprehensive, multivariable analyses incorporating multiple exposures and a spectrum of oral health outcomes have been conspicuously absent in the Hungarian literature. This lack of population-based evidence constitutes a major gap, impeding the development of targeted, data-driven oral health prevention strategies.

Thus, the primary aim of this study was to investigate the association of beverage consumption covering sugar-sweetened beverages, energy drinks, dairy, and alcohol with oral health outcomes. We also evaluated the influence of sociodemographic and behavioral factors as independent variables in a nationally representative sample of Hungarian adults.

## 2. Materials and Methods

### 2.1. Study Population and Data Source

This study utilized data from the third wave (2019) of the Hungarian European Health Interview Survey (EHIS), a nationally representative cross-sectional survey of adults aged 15 years and older, conducted by the Hungarian Central Statistical Office [11]. The survey’s sampling and data collection procedures have been described in detail elsewhere [12,13,14].

### 2.2. Variable Definitions and Measurement

The original survey sample consisted of 5603 responses. All respondents younger than 18 years of age were excluded, resulting in a final analytic sample of 5425 adults. Sociodemographic variables were categorized as follows: sex (male, female); age group (18–34, 35–64, 65+); educational attainment (primary, secondary, tertiary); employment status (employed, unemployed); area of residence (urban, rural); and self-reported financial status (good, average, bad). Oral health outcomes were operationalized as follows: presence of active caries (dichotomized as no/yes), presence of filled teeth (no/yes), gum bleeding when brushing teeth (no/yes), and mobile teeth (no/yes), all based on self-report; number of missing teeth, excluding teeth extracted for orthodontic reasons and wisdom teeth (classified as 0, 1–5, 6–19, or 20+); and self-perceived oral health, rated as good, average, or poor. Smoking status was categorized based on the question, “Which statement best describes your smoking behavior?” with response options never smoked, quit, or currently smokes. Alcohol consumption frequency over the past 12 months was classified as abstinent, less than once a month, 1–3 times/month, 1–4 times/week, or daily/almost daily. Dietary exposures included the frequency of dairy product consumption (“How often do you consume milk or dairy products?”), 100% fruit juice, sugar-sweetened soda drinks, energy drinks, and sports drinks; each was categorized as rarely or never, weekly, or daily. Variable coding was consistent with previous analyses of the Hungarian EHIS 2019 dataset, as detailed in references [15,16].

### 2.3. Statistical Analysis

All analyses were conducted in Stata version 18 [17]. Descriptive statistics were computed as counts and proportions for all variables. Bivariate associations between categorical predictors and oral health outcomes were assessed using Pearson’s chi-squared and Fisher’s exact tests. For multivariable analysis, binary and ordinal logistic regression models were constructed as appropriate to estimate odds ratios (ORs) and corresponding 95% confidence intervals (CIs) for each outcome. Multicollinearity among covariates was evaluated using the variance inflation factor (VIF), with a threshold of VIF > 10 indicating problematic collinearity. Goodness-of-fit for binary logistic models was assessed using the Hosmer–Lemeshow test. Model discrimination and classification accuracy were further evaluated using confusion matrices, with calculations of sensitivity, specificity, positive predictive value, and negative predictive value. Sensitivity analyses were performed to examine the robustness of the main findings to alternate model specifications and subpopulation restrictions. All statistical tests were two-sided, with *p*-values < 0.05 considered indicative of statistical significance. No missing data imputation was conducted; analyses relied on complete-case data only.

## 3. Results

### 3.1. Bivariate Associations Between Oral Health Indicators and Covariates

Active dental caries was significantly more prevalent among males (32.3%) than females (25.6%), and highest in the 18–34 age group (36.0%), with prevalence decreasing to 16.6% in those 65 years or older (all *p* < 0.001). The highest rate was observed in individuals with primary education (36.4%), among the employed (32.8%), rural residents (35.0%), and those reporting bad financial status (37.8%) (all *p* < 0.001). Current smokers had notably higher prevalence (40.7%) compared to never (23.6%) and former smokers (25.8%, *p* < 0.001). Both daily soda (43.8%) and daily energy drink (51.9%) consumption were associated with higher caries (*p* < 0.001), as was lower frequency of dairy intake (*p* < 0.001). No significant associations were observed for alcohol, juice, or sport drink consumption (Table 1).

The prevalence of filled teeth due to caries was highest among those aged 35–64 (76.0%) and 15–34 (72.6%), but lowest in the 65+ group (44.5%, *p* < 0.001). Higher rates were observed in individuals with tertiary education (83.7%), the employed (79.3%), and urban residents (68.7%) (all *p* < 0.001). Financial status was significant, with those reporting good financial status having a prevalence of 70.9% (*p* < 0.001). Never smokers had the highest prevalence of filled teeth (69.4%), while current smokers showed the lowest (61.6%, *p* < 0.001). Significant associations were also seen with higher frequency of dairy (68.2% for 4+/week), juice (69.9% for weekly), and soda (72.1% for weekly) consumption. Sport drink consumption showed a significant difference (*p* = 0.008), while energy drink consumption was not significant (*p* = 0.573). Sex was not significantly associated with filled teeth (*p* = 0.107) (Table 1).

Statistically significant associations were noted for gum bleeding upon toothbrushing across sex (higher in females, 17.4% vs. 14.6%, *p* = 0.005) and age group (prevalence highest in 18–34 years at 21.7%, lowest in 65+ at 10.6%, *p* < 0.001). There was no significant difference by education (*p* = 0.1). Gum bleeding was more common in employed individuals (17.3%, *p* = 0.015) and showed a trend by financial status (*p* = 0.009), with the highest prevalence among those with “bad” status (19.7%). Smoking status was also significant (*p* = 0.016), with higher rates among those who quit (19.0%) compared to never smokers (15.5%). Dairy frequency (rarely/never: 20.9%), juice frequency (rarely/never: 17.4%), soda (daily: 25.0%), and energy drink (daily: 29.5%) intake were all positively associated with gum bleeding (all *p* < 0.05) (Table 2).

Prevalence of mobile teeth was significantly higher in older adults (10.1% in 65+ vs. 3.8% in 18–34, *p* < 0.001), in those with lower education (primary: 12.0%, tertiary: 4.4%, *p* < 0.001), and in the unemployed (9.9% vs. 6.8% employed, *p* < 0.001). Rural residents (10.6%) had a higher prevalence than urban residents (7.2%, *p* < 0.001). “Bad” financial status was associated with the highest prevalence (15.9%, *p* < 0.001). Smokers (10.3%) had a higher prevalence than never smokers (7.4%, *p* = 0.006). Alcohol use was inversely associated (*p* < 0.001). Low dairy frequency (rarely/never: 13.9%, *p* < 0.001), rarely/never juice consumption (9.5%, *p* < 0.001), and daily energy drink intake (10.8%, *p* = 0.03) were also associated with mobile teeth. No significant associations were found with sex, soda frequency, or sport drink frequency (Table 2).

Marked gradients were found in the number of missing teeth across age, education, employment, residence, financial status, smoking, and dietary behaviors (all *p* < 0.001). The prevalence of having no missing teeth was highest in the 15–34 age group (12.1%), declining to 3.6% in 35–64 and 2.0% in 65+ years, while 44.4% of those 65+ had 20 or more missing teeth (*p* < 0.001). Among those with tertiary education, 8.1% had no missing teeth versus 2.2% among those with primary education; the proportion with 20+ missing teeth was 31.9% in primary versus 8.7% in tertiary education (*p* < 0.001). Employed respondents more frequently had no missing teeth (5.5%) compared to unemployed (2.9%), while the unemployed had a higher prevalence of 20+ missing teeth (36.3% vs. 7.1%, *p* < 0.001). Rural residents had more than 20+ missing teeth (24.5%) compared to urban residents (20.5%, *p* < 0.001). Respondents with good financial status more often had no missing teeth (6.6%) compared to those with bad status (2.9%, *p* < 0.001). Never smokers had the highest proportion with no missing teeth (4.7%) and the lowest with 20+ missing (20.6%), compared to quitters (26.6%) and current smokers (20.8%, *p* < 0.001). Among those consuming dairy ≥ 4x/week, 4.0% had no missing teeth and 21.2% had 20+ missing, compared to rarely/never (4.9% and 29.6%, respectively; *p* < 0.001). Daily soda and energy drink consumers had higher proportions with 6–19 or 20+ missing teeth than those rarely/never consuming them (soda daily: 23.7% and 19.9%; rarely/never: 29.0% and 25.0%; *p* < 0.001 for both) (Table 3).

Self-perceived oral health also varied significantly by most variables (all *p* < 0.001, sex *p* = 0.141). Good oral health was reported by 68.1% of respondents aged 15–34 versus 27.8% of those 65+ (*p* < 0.001). Among those with tertiary education, 60.7% reported good oral health and only 8.1% poor oral health, compared to 32.8% and 32.6%, respectively, among those with primary education (*p* < 0.001). Employed individuals more often rated their oral health as good (54.9%) versus unemployed (35.0%, *p* < 0.001). Good oral health was more frequent in urban (47.9%) than rural areas (39.7%, *p* < 0.001), and in those with good financial status (61.0%) compared to bad (24.4%, *p* < 0.001). Never smokers more frequently reported good oral health (50.5%) compared to current (40.3%) or former smokers (37.8%, *p* < 0.001). The prevalence of good oral health was highest in those with dairy ≥ 4x/week (46.8%), and lowest in those rarely/never consuming dairy (39.9%, *p* < 0.001). Frequent juice consumers (weekly) reported good oral health more often (54.8%) than those rarely/never (41.2%, *p* < 0.001), while daily sport drink or energy drink use was associated with a lower prevalence of good oral health (sport drink daily: 62.5% good, 6.3% poor; energy drink daily: 46.8% good, 27.3% poor, *p* < 0.001 for both) (Table 3).

### 3.2. Multiple Regression Modeling of Oral Health Outcomes

Across models, female sex was associated with higher odds of having filled teeth (OR = 1.34, 95% CI: 1.16–1.56, *p* < 0.001) and gum bleeding when brushing (OR = 1.55, 95% CI: 1.29–1.85, *p* < 0.001). Older age groups (65+) were less likely to have filled teeth (OR = 0.40, 95% CI: 0.32–0.50, *p* < 0.001), gum bleeding (OR = 0.37, 95% CI: 0.28–0.49, *p* < 0.001), or dental caries. Higher educational attainment was strongly associated with having filled teeth (tertiary: OR = 3.45, 95% CI: 2.81–4.22, *p* < 0.001), but was protective against gum bleeding (secondary: OR = 0.78, 95% CI: 0.65–0.95, *p* = 0.013) and dental caries (tertiary: OR = 0.56, 95% CI: 0.46–0.69, *p* < 0.001).

Employment was linked with more favorable outcomes, as employed individuals had higher odds of having filled teeth (OR = 2.06, 95% CI: 1.74–2.45, *p* < 0.001). Urban residence increased the odds of having filled teeth (OR = 1.17, 95% CI: 1.02–1.35, *p* = 0.029), but reduced the likelihood of decay (OR = 0.82, 95% CI: 0.71–0.94, *p* = 0.005). Good financial status was associated with lower odds of tooth decay (OR = 0.66, 95% CI: 0.56–0.77, *p* < 0.001), and gum bleeding (OR = 0.83, 95% CI: 0.69–0.99, *p* = 0.041) (Table 4).

Smoking showed divergent effects: current smokers were less likely to have filled teeth (OR = 0.62, 95% CI: 0.53–0.73, *p* < 0.001) or gum bleeding (OR = 0.68, 95% CI: 0.56–0.83, *p* < 0.001), but more likely to have dental caries (OR = 1.42, 95% CI: 1.21–1.66, *p* = 0.001). Alcohol consumption of 1–3 times per month and 1–4 times per week was linked with increased odds of filled teeth (OR = 1.31, 95% CI: 1.08–1.59, *p* = 0.007; OR = 1.44, 95% CI: 1.16–1.79, *p* = 0.001), while higher odds of gum bleeding were reported when frequency of alcohol consumption was 1–4 times per week (OR = 1.38, 95% CI: 1.07–1.77, *p* = 0.014) (Table 4).

Dietary habits also contributed: higher dairy consumption (4+ times/week) was associated with increased odds of having filled teeth (OR = 1.59, 95% CI: 1.29–1.96, *p* < 0.001), and was protective against gum bleeding (OR = 0.76, 95% CI: 0.59–0.96, *p* = 0.023). Weekly fresh juice intake reduced the odds of gum bleeding (OR = 0.62, 95% CI: 0.51–0.76, *p* < 0.001) and dental caries (OR = 0.84, 95% CI: 0.72–0.99, *p* = 0.042). More frequent soda and energy drink consumption was linked with higher odds of gum bleeding (daily soda: OR = 1.94, 95% CI: 1.53–2.47, *p* < 0.001; daily energy drink: OR = 1.56, 95% CI: 1.01–2.42, *p* = 0.047), and they were also linked to higher odds of having tooth decay (daily soda: OR = 1.57, 95% CI: 1.27–1.94, *p* < 0.001; daily energy drink: OR = 1.55, 95% CI: 1.03–2.31, *p* = 0.034). Daily consumption of sports drinks reduced the likelihood of tooth decay (OR = 0.24, 95% CI: 0.06–0.94, *p* = 0.040) (Table 4).

Across multivariable models, older age, lower education, rural residence, and poor financial status consistently predicted worse oral health outcomes. For tooth mobility (binary logistic regression), higher age was associated with increased odds (65+ vs. 18–34: OR = 1.90, 95% CI: 1.24–2.90, *p* < 0.001), while tertiary education was protective (OR = 0.55, 95% CI: 0.39–0.77, *p* = 0.001). Urban residence reduced the odds of tooth mobility (OR = 0.79, 95% CI: 0.64–0.98, *p* = 0.034), as did good financial status (OR = 0.84, 95% CI: 0.64–1.10, *p* = 0.198; not significant), while poor financial status was a risk factor (OR = 1.77, 95% CI: 1.36–2.31, *p* < 0.001). Employment was not significantly associated (OR = 0.80, 95% CI: 0.61–1.05, *p* = 0.109). Daily/almost daily alcohol use increased the odds of tooth mobility (OR = 1.47, 95% CI: 1.02–2.11, *p* = 0.039). High frequency of dairy intake was associated with lower odds of tooth mobility (OR = 0.63, 95% CI: 0.47–1.85, *p* = 0.002) as was weekly intake of fresh juice (OR = 0.71, 95% CI: 0.53–0.94, *p* = 0.017) (Table 5).

For number of missing teeth (ordinal logistic regression: 0, 1–5, 6–19, 20+), older age showed a strong association (35–64 vs. 18–34: OR = 6.14, 95% CI: 4.85–7.77, *p* < 0.001), and lower education increased odds of more missing teeth (secondary: OR = 0.52, 95% CI: 0.45–0.60, *p* < 0.001; tertiary: OR = 0.30, 95% CI: 0.25–0.36, *p* < 0.001, both vs. primary). Urban residence (OR = 0.79, 95% CI: 0.69–0.90, *p* = 0.001), employment (OR = 0.49, 95% CI: 0.42–0.58, *p* < 0.001), and good financial status (OR = 0.86, 95% CI: 0.74–0.99, *p* < 0.001) were protective. Smoking (current: OR = 1.81, 95% CI: 1.55–2.10, *p* < 0.001) increased the odds of having more teeth missing. Dairy intake reduced the odds of having missing teeth with ORs for once per week (OR = 0.63), 2–3 times per week (OR = 0.79), and more than four times per week (OR = 78), all showing significant associations. Fresh juice intake on a weekly basis significantly reduced the odds of having more teeth missing (OR = 0.83, 95% CI: 0.71–0.96, *p* = 0.015) (Table 5).

For self-perceived oral health (ordinal logistic regression: good, average, poor), older age (65+ vs. 18–34: OR = 3.90, 95% CI: 3.19–4.78, *p* < 0.001), lower education (tertiary: OR = 0.47, 95% CI: 0.40–0.55, *p* < 0.001), rural residence (urban: OR = 0.75, 95% CI: 0.67–0.85, *p* < 0.001), and poor financial status (OR = 1.78, 95% CI: 1.51–2.11, *p* < 0.001) were again key predictors. Being employed (OR = 0.66, 95% CI: 0.57–0.76, *p* < 0.001), good financial status (OR = 0.60, 95% CI: 0.52–0.62, *p* < 0.001), and frequent juice consumption were associated with lower odds of reporting poor oral health. Smoking (current: OR = 1.76, 95% CI: 1.53–2.02, *p* < 0.001) and higher alcohol intake were also significant risk factors. Daily soda intake appeared to significantly increase the odds of one perceiving their oral health poorly (OR = 1.32, 95% CI: 1.10–1.59, *p* = 0.004) (Table 5).

## 4. Discussion

A variety of factors can influence oral health; therefore, identifying key risk factors is essential for the development of effective preventive strategies. This study aimed to examine the impact of sociodemographic factors, lifestyle factors, and different types of beverage consumption on oral health in the Hungarian population.

The study revealed that women had significantly lower odds of having active caries but higher odds of having filled teeth due to decay and experiencing gum bleeding, compared to men. The lower prevalence of active caries and the higher number of filled teeth observed among women are consistent with previous studies suggesting that women typically maintain better oral hygiene practices. Men generally demonstrate lower adherence to oral hygiene practices and are less likely to seek regular dental care [18]. As a result, the female sex may be associated with a lower risk of active dental caries, while it may also be linked to a higher likelihood of gingival bleeding. The increased prevalence of gingival bleeding observed in women is likely associated with hormonal influences; during periods of hormonal fluctuation such as puberty, pregnancy, oral contraceptive use, and postmenopause, the periodontium is subject to a heightened inflammatory response to dental plaque due to the effects of female sex hormones. Estrogen primarily contributes to alterations in vascular structure and function, while progesterone promotes the release of inflammatory mediators, including prostaglandins and cytokines. These hormonal effects collectively increase susceptibility to gingival bleeding [19,20].

The 65-year and older age group had lower odds of having active dental caries, a higher number of filled teeth, and reduced gingival bleeding. Our data suggested that the reduced prevalence of gingival bleeding in this age group does not necessarily indicate better periodontal health. In advanced stages of periodontal disease, tooth loss becomes common, and instead of typical inflammatory signs such as bleeding, gingival recession and edentulism are more frequently observed [21]. This is further supported by our findings, which show that tooth loss is significantly more prevalent among individuals aged 65 and older compared to those aged 18–34 and 35–64. The highest rate of tooth mobility was observed in the 35–64 age group, potentially reflecting an active, yet not end-stage, phase of periodontal disease. Both tooth mobility and tooth loss can be interpreted as consequences of prolonged, chronic inflammatory processes [22]. The elderly exhibit several risk factors, including increased vulnerability to infections and oral diseases [23]. Although some older individuals perceive dental diseases as a natural part of aging, which may help mitigate the psychological impact of age-related oral health decline [24], previous research has demonstrated a negative correlation between aging and self-reported oral health. In line with the cross-sectional study by Pengpid et al. [25], we found that older age was associated with higher odds of poor self-rated oral health. Specifically, individuals aged 65 and above had nearly four times the odds of reporting poorer oral health compared to those aged 18–34. Additionally, those in the 35–64 age group showed a 2.45 times higher odds ratio. These findings suggest that self-perceived oral health in different age groups may serve as a predictor of oral health outcomes and highlight the importance of promoting health awareness among older populations.

Educational level was significantly associated with oral health outcomes. Compared to individuals with primary education, those with secondary education had significantly lower odds of active caries, with the lowest odds observed among individuals with tertiary education. Regarding the number of filled teeth due to decay, individuals with secondary education had significantly higher odds, and those with tertiary education showed the highest odds relative to the primary education group. In the case of gingival bleeding, secondary education was associated with a statistically significant reduction in odds, while no significant association was found for tertiary education in comparison to those with only primary education, although a slight reduction was also observed in this group. Compared to participants with primary education, those with secondary education had significantly lower odds of tooth mobility and fewer missing teeth, with the lowest values observed among those with tertiary education. Higher educational attainment might be linked to improved knowledge of caries, periodontal disease, and oral hygiene preventive measures. Accordingly, it is reasonable to assume that individuals with more advanced education are more likely to be familiar with proper tooth brushing/dental floss techniques and to recognize the importance of regular dental check-ups [26]. As a result, individuals with higher educational attainment are more likely to have dental restorations, since carious lesions are detected and treated in a timely manner during regular dental visits, and they tend to exhibit lower prevalence of gingival bleeding, tooth mobility, and tooth loss associated with periodontal disease. Better oral health outcomes were associated with higher education levels. Accordingly, individuals with secondary education had significantly lower odds of poor self-perceived oral health compared to those with only primary education, while the lowest odds were observed among those with tertiary education. These findings support the association between higher levels of education and better oral health self-assessment, and are consistent with the cross-sectional analysis by Bof de Andrade et al., which emphasizes the importance of evaluating various socioeconomic indicators [27].

Not only educational attainment but also employment status can be an important determinant of oral health. In our study, employed individuals showed significantly higher odds of having a greater number of filled teeth and lower odds of missing teeth. Several epidemiological studies support that regular dental care is more common among employed individuals. Data suggest that employment facilitates regular dental check-ups. According to health survey data in Finland, employed individuals had more frequent dental fillings, while unemployed persons exhibited a higher number of missing and decayed teeth and teeth with periodontal pockets [28]. Employed individuals subjectively assessed their oral health more positively compared to unemployed participants. These results are consistent with the findings of Hakeberg et al., who reported that unemployed individuals were more likely to rate their oral health as poor. This supports the identification of vulnerable population groups [29].

The results of this study indicate that urban residency is associated with lower odds of active dental caries, tooth mobility, and tooth loss, as well as higher odds of possessing filled teeth when compared to rural residents. This pattern suggests that individuals residing in urban areas experience a lower prevalence of untreated oral diseases and benefit from improved access to restorative dental services. Urban populations are also more likely to participate in preventive dental programs and utilize dental care resources compared to those living in rural settings [30]. As a result, we found that urban residents tend to rate their oral health more positively. This finding is in line with a cross-sectional study by Costa et al., which showed that factors such as access to care and lifestyle influence both self-perceived and clinically assessed oral health among elderly individuals, with rural populations showing the poorest outcomes [31].

Good financial status appeared to be associated with lower levels of dental caries, gingival bleeding, and tooth loss. Individuals with favorable financial status demonstrated significantly lower odds of active dental caries, gingival bleeding, and missing teeth compared to those with an average financial situation. In contrast, individuals with poor financial status displayed significantly higher odds of tooth mobility compared to those with average financial conditions. Low income may be a financial barrier to dental care [32], whereas higher income levels are typically linked to improved access to healthcare services. Individuals with greater financial stability may be more likely to engage in preventive care, seek timely treatment and adopt a health-conscious lifestyle. During periods of economic crisis, oral health often becomes a lower priority and receives diminished attention from individuals affected by financial hardship [33]. A systematic review by Cianetti et al., which examined the relationship between sociodemographic disparities and oral diseases, reached conclusions consistent with our own findings: Socioeconomically disadvantaged individuals were found to be more susceptible to tooth decay and periodontal disease, often accompanied by tooth loss, compared to non-vulnerable populations. The authors reported that low-income individuals have approximately twice the risk of developing dental caries compared to those with a more stable or higher income. Moreover, a higher prevalence of periodontitis, as well as more frequent partial tooth loss or complete edentulism, can be observed among individuals with low income [34]. Unfavorable oral health conditions are reflected in individuals’ self-perception: those with disadvantaged financial status were more likely to report poor oral health compared to those with average financial status, while individuals with good financial standing were less likely to assess their oral health as poor. This aligns with the findings of Molarius et al., who reported that financially secure individuals within the studied population tended to assess their oral health more positively [35].

In addition to sociodemographic factors, behavioral variables such as smoking and beverage consumption patterns appear to have a significant impact on oral health. Analyzing smoking habits in the examined population, we observed significantly higher odds of active caries and lower odds of filled teeth among smokers compared to non-smokers. Thus, smoking may be associated with an increased risk of developing dental caries and a lower likelihood of having dental fillings. The adverse effects of smoking on oral health may include reduced salivary flow, impaired buffering capacity of saliva, and lowered levels of salivary immunoglobulin A (IgA) [36]. Cigarette smoking contributes to oral dysbiosis by altering the diversity and functional potential of the oral microbiota, contributing to the development of dental caries [37]. Previous studies, including a systematic review and meta-analyses by Jiang et al., have also found that smoking is associated with higher numbers of decayed teeth [38]. Smokers are also less likely to attend regular dental check-ups [39]; according to the analysis by Drilea et al., a smaller proportion of current smokers reported visiting a dentist (32.9%) compared to non-smokers (45.0%) [40], which may explain the lower number of filled teeth observed among individuals who smoke. Current smokers exhibited significantly lower odds of gum bleeding, whereas those who quit smoking displayed higher odds compared to never-smokers. During smoking, nicotine causes strong vasoconstriction, leading to reduced blood flow in the gums. This can mask bleeding, even when inflammation is present. Upon quitting smoking, blood circulation improves, revealing the previously hidden inflammation and resulting in a higher occurrence of gum bleeding. In the study conducted by Nair et al., the prevalence of gingival bleeding among individuals who quit smoking increased from 16% to 32% within 4 to 6 weeks. This finding suggests that smoking suppresses the clinical signs of inflammation, which reappear after cessation [41]. Furthermore, both current and former smokers exhibited significantly increased odds of tooth loss compared to individuals who had never smoked. Cigarette smoking may contribute to the progression of periodontal disease through several mechanisms. Prior research indicates that components in tobacco smoke can favor the growth of plaque-associated anaerobic bacteria, potentially disrupting the balance of the oral microbiome [42]. Additionally, smoking has been linked to reduced antioxidant capacity in saliva, which may impair the host’s defense against oxidative stress [43]. As a result, free radicals generated during the immune response to bacterial presence can harm periodontal tissues, ultimately increasing the risk of tooth loss associated with periodontal disease [44]. Furthermore, smoking may exert harmful effects on bone metabolism. Evidence suggests that nicotine can lead to decreased bone mineral density and content, either by enhancing the secretion of bone-resorbing mediators [45] or by impairing calcium absorption in the gastrointestinal tract [46]. These physiological effects of smoking may not only lead to clinically detectable outcomes but can also shape how individuals perceive their own oral health status. Both current and former smokers demonstrated significantly higher odds of reporting poorer self-perceived oral health, aligning with findings from previous studies [39].

Our results indicated that, compared to abstainers, individuals who reported alcohol consumption less than once per month had significantly lower odds of presenting with active dental caries. Participants consuming alcohol 1–3 times per month or 1–4 times per week exhibited significantly higher odds of having teeth filled due to dental decay. Alcohol consumption at a frequency of 1–4 times per week was associated with significantly increased odds of gingival bleeding. Daily alcohol use was linked to significantly higher odds of tooth mobility. Consuming alcohol less than once a month may be considered a protective factor, whereas more frequent consumption is associated with progressively increasing risk. Various types of alcoholic beverages available on the market are known to reduce salivary flow. Many of these drinks contain sugars that support acid production by pathogenic microorganisms in the oral cavity, creating an acidic environment that facilitates enamel demineralization and leads to damage of both teeth and gingival tissues. Chronic alcohol consumption has been associated with multiple oral health issues, including dental caries, and is frequently linked with tobacco use. Regular intake of alcohol may disrupt the composition of the oral microbiome, favoring the proliferation of pathogenic species and thereby contributing to the development of periodontal disease [47]. Previous literature has clearly demonstrated that alcohol use increases the risk of periodontal diseases in a dose-dependent manner. According to a meta-analysis conducted by Wang et al., each additional 1 g/day of alcohol consumption is associated with approximately a 0.4% increase in the risk of periodontitis [48]. Such oral conditions may negatively impact comfort, aesthetics, and pain levels, ultimately leading to reduced overall satisfaction [49]. In our study, in cases of daily/almost daily alcohol drinkers, perceiving their oral health as “poor” was significantly higher compared to non-drinkers among respondents. Interestingly, individuals who consumed alcohol 1–3 times per month or 1–4 times per week demonstrated lower odds of having missing teeth. This finding is consistent with some previous studies, where moderate alcohol consumption has been associated with fewer missing teeth among both middle-aged [50] and older adults [51]; however, other studies have reported a positive association between alcohol intake and tooth loss [52]. The overall evidence remains inconsistent; further research is required to clarify the underlying causal mechanisms.

We found significantly higher odds of having filled teeth among individuals who consume dairy products at least once a week, compared to those who rarely or never consume them, and the odds of gum bleeding were significantly lower among those who consumed dairy at least 2–3 times per week. In addition, significantly lower odds of tooth mobility were observed among participants who consumed dairy at least four times per week. Furthermore, all individuals who consumed dairy had significantly lower odds of missing teeth compared to those who rarely or never did. Regular dairy consumption might appear as a risk factor for dental fillings, but as a protective factor in relation to gingival bleeding. A population-based study reported that larger quantities of milk were associated with an increased risk of caries, particularly among women [53]. This aligns with our findings that individuals who consume more dairy may experience caries more frequently, which in turn requires more fillings. On the other hand, while the natural sugar (lactose) in milk can be metabolized by cariogenic bacteria, producing acid and potentially leading to enamel demineralization and restorative treatments such as fillings, it is considered by other studies to be less acidogenic and cariogenic than other sugars such as sucrose [54,55]. Therefore, the higher number of filled teeth observed in this study may be more closely linked to the frequency of additional sugar intake and various dietary or lifestyle habits, rather than to milk consumption itself. In the study by Utsunomiya et al., the consumption of milk, cheese, and yogurt was associated with a 21%, 26%, and 35% reduction, respectively, in the odds of dental caries prevalence. In contrast, intake of other dairy-based products such as probiotic milk, ice cream, and custard pudding was associated with a 2.3-fold increase in the odds of developing caries [56]. The beneficial association observed with milk, cheese, and yogurt may be attributable to their content of bioactive peptides and minerals, particularly calcium and phosphorus. These components possess anti-inflammatory properties and contribute to maintaining gingival tissue integrity and supporting overall oral health [57,58]. In addition to the anti-inflammatory properties, dairy products such as milk and cheese promote oral health by increasing saliva flow and buffering capacity, enhancing enamel remineralization through calcium and phosphate, and inhibiting the adherence of cariogenic bacteria such as *Streptococcus mutans*. Casein, the primary protein found in milk, may help protect tooth enamel by forming a protective film on its surface, which could reduce the susceptibility of the enamel to acid damage and the development of dental caries [59,60].

In our study, individuals who consumed juice on a weekly basis had significantly reduced odds of active caries, gingival bleeding, tooth mobility, and tooth loss compared to those who never consume juice. This finding may suggest that moderate consumption, particularly when associated with meals, can have a beneficial effect on gingival health. Fruit juices (e.g., citrus fruits or tomato) are a significant source of vitamin C and also contain antioxidants, which help prevent gingivitis and promote collagen synthesis [61,62]. Reflecting the beneficial effects of moderate juice consumption, participants who consumed juice weekly reported significantly better oral health outcomes compared to those who never consume juice.

Our analysis revealed that individuals who consume soda have significantly higher odds of active caries and gingival bleeding compared to those who abstain from soda consumption. In the 2019 Hungarian EHIS questionnaire, the term “soda” referred specifically to sugar-sweetened carbonated soft drinks; therefore, our findings pertain exclusively to this beverage type. Carbonated soft drinks have an acidic pH that can dissolve tooth enamel, and their high sugar content promotes the growth of cariogenic bacteria and the formation resulting in a greater risk of caries [63]. Research indicates that there is a statistically significant positive association between the frequency of sugar intake and the severity of plaque-induced gingivitis in various populations, including children. This connection is important given that excessive sugar consumption can lead to increased plaque formation and subsequent gingival inflammation, highlighting the role of dietary choices in oral health [64]. According to a systematic review by Gupta et al., the consumption of sugar-sweetened beverages may contribute to increased gingival bleeding, which can lead to the onset of gingivitis and subsequently elevate the risk of developing periodontitis. Thus, the consumption of added sugars, including sugar-sweetened beverages, should be taken into account as a potential risk factor in gingival and periodontal assessments [65]. Respondents who reported daily consumption of carbonated drinks were more likely to rate their oral health as “poor” compared to non-consumers, which aligns with previous findings. Sim et al. also reported that the consumption of sugar-sweetened beverages negatively influenced adolescents’ subjective perception of oral health; those who consumed sugar-sweetened beverages were more likely to report self-perceived oral symptoms [66].

A similar association was observed among energy drink consumers: individuals who consumed energy drinks daily exhibited a significantly higher likelihood of active caries and gingival bleeding compared to those who avoided such beverages. Energy drinks are characterized by low pH and high carbohydrate content. Frequent and excessive intake of these beverages contributes to dental erosion, increases the risk of dental caries, and may also compromise the integrity of restorative materials [67]. These findings suggest that sugar-sweetened and acidic beverages, including energy drinks, may collectively contribute to unfavorable oral health outcomes [63,64,65,66]. Beyond caries and periodontal outcomes, excessive intake of acidic and sugary beverages may also contribute to mucosal irritation and lesion development, especially in individuals with orthodontic appliances, as highlighted by Manuelli et al. [68].

Finally, we examined the impact of sport drink consumption on oral health and an unexpected finding was observed: individuals who consumed sports drinks daily had significantly lower odds of active caries compared to those who never or rarely consumed them, suggesting a reduced likelihood of developing dental caries in the studied population. Hooper et al. found that specialized formulations designed for athletes and enriched with calcium reduced the erosive potential of these beverages and promoted enamel remineralization, thereby contributing to greater enamel stability. Additionally, when appropriately pH-adjusted, such drinks were less erosive to tooth enamel [69]. These previous studies might support our results; however, the observed lower risk of caries may also be attributed to greater health awareness and more consistent oral hygiene habits among athletes. Individuals who consume sports drinks daily may be more physically active and health-conscious, thus engaging in better oral hygiene practices, regular dental visits, or overall healthier behaviors that mitigate the potential harmful effects of these beverages. Further research is needed to better understand the role of possible confounding factors.

### Strengths and Limitations

A major strength of this study is the use of nationally representative EHIS 2019 data, which enables robust, generalizable conclusions for the Hungarian adult population. The large sample size and comprehensive inclusion of sociodemographic, behavioral, and dietary variables allowed for detailed multivariable analyses of oral health outcomes. Use of standardized EHIS methodology ensures comparability with international research.

However, the cross-sectional design precludes causal inference and may be subject to reverse causation. All variables, including oral health outcomes and exposures, were self-reported, introducing potential for recall and reporting bias. The dietary frequency measurements were also based on self-reports, without information on portion size, which may affect the accuracy of exposure assessment. Residual confounding from unmeasured factors cannot be excluded. Finally, certain oral health outcomes were not clinically validated, which may limit the precision of some findings. Future prospective or interventional studies are needed to strengthen the evidence for observed associations.

## 5. Conclusions

These results provide strong evidence that modifiable lifestyle factors play a decisive role in shaping oral health outcomes, regardless of sociodemographic background. This evidence reinforces that oral health is largely shaped by choices that can be changed. Public health interventions that encourage healthier dietary patterns, such as increasing dairy intake and reducing the consumption of sugar-sweetened and acidic beverages, together with strategies to reduce smoking and excessive alcohol use, are likely to result in significant improvements in oral health at the population level. Implementing community-level strategies that encourage regular consumption of dairy products and fresh juice, while reducing intake of sugar-sweetened and acidic beverages, directly addresses key dietary risk factors. Coupling these with comprehensive smoking cessation and alcohol reduction programs can further decrease the burden of oral disease. Integrating routine oral health screening into primary healthcare settings would facilitate early identification and intervention, ensuring that modifiable risk factors are addressed before significant disease develops. Collectively, these measures offer a practical pathway toward significant and sustainable improvements in population oral health.

## Figures and Tables

**Table 1:** Distribution of active dental caries and filled teeth by sociodemographic and behavioral characteristics (row percentages shown).

Variable	Category	No Active Caries (n, %)	Has Active Caries (n, %)	*p*-Value	No Filled Teeth (n, %)	Has Filled Teeth (n, %)	*p*-Value
Sex	Male	1597 (67.67)	763 (32.33)	**<0.001**	853 (35.10)	1577 (64.90)	0.107
	Female	2071 (74.42)	712 (25.58)		950 (33.00)	1929 (67.00)	
Age group	18–34	670 (63.99)	377 (36.01)	**<0.001**	298 (27.36)	791 (72.64)	**<0.001**
	35–64	1715 (67.07)	842 (32.93)		636 (23.96)	2018 (76.04)	
	65+	1283 (83.37)	256 (16.63)		869 (55.49)	697 (44.51)	
Education	Primary	1442 (63.58)	826 (36.42)	**<0.001**	1118 (48.80)	1173 (51.20)	**<0.001**
	Secondary	1306 (75.45)	425 (24.55)		485 (27.05)	1308 (72.95)	
	Tertiary	920 (80.42)	224 (19.58)		200 (16.33)	1025 (83.67)	
Employment	Unemployed	1889 (75.65)	608 (24.35)	**<0.001**	1230 (48.35)	1314 (51.65)	**<0.001**
	Employed	1779 (67.23)	867 (32.77)		573 (20.72)	2192 (79.28)	
Residence	Rural	1088 (65.03)	585 (34.97)	**<0.001**	674 (39.60)	1028 (60.40)	**<0.001**
	Urban	2580 (74.35)	890 (25.65)		1129 (31.30)	2478 (68.70)	
Financial status	Average	2046 (69.95)	879 (30.05)	**<0.001**	1068 (35.38)	1951 (64.62)	**<0.001**
	Good	1163 (77.69)	334 (22.31)		447 (29.06)	1091 (70.94)	
	Poor	388 (62.18)	236 (37.82)		261 (40.59)	382 (59.41)	
Smoking status	Never	2082 (76.38)	644 (23.62)	**<0.001**	862 (30.63)	1952 (69.37)	**<0.001**
	Quit	727 (74.18)	253 (25.82)		380 (37.36)	637 (62.64)	
	Smokes	824 (59.32)	565 (40.68)		548 (38.43)	878 (61.57)	
Alcohol	Abstinent	1090 (70.69)	452 (29.31)	0.3	663 (42.34)	903 (57.66)	**<0.001**
	<1/month	904 (73.20)	331 (26.80)		411 (32.39)	858 (67.61)	
	1–3/month	739 (70.45)	310 (29.55)		290 (26.51)	804 (73.49)	
	1–4/week	577 (72.13)	223 (27.88)		227 (26.96)	615 (73.04)	
	Daily/almost daily	322 (68.51)	148 (31.49)		198 (40.57)	290 (59.43)	
Dairy frequency	Rarely/never	367 (67.84)	174 (32.16)	**<0.001**	247 (44.34)	310 (55.66)	**<0.001**
	1/week	201 (64.84)	109 (35.16)		110 (34.70)	207 (65.30)	
	2–3/week	701 (68.39)	324 (31.61)		377 (35.43)	687 (64.57)	
	4+/week	2378 (73.35)	864 (26.65)		1063 (31.81)	2279 (68.19)	
Juice frequency	Rarely/never	2417 (70.38)	1017 (29.62)	0.086	1265 (35.67)	2281 (64.33)	**0.001**
	Weekly	914 (73.12)	336 (26.88)		389 (30.11)	903 (69.89)	
	Daily	294 (74.06)	103 (25.94)		132 (32.75)	271 (67.25)	
Soda frequency	Rarely/never	2578 (75.98)	815 (24.02)	**<0.001**	1230 (35.16)	2268 (64.84)	**<0.001**
	Weekly	740 (64.80)	402 (35.20)		330 (27.90)	853 (72.10)	
	Daily	316 (56.23)	246 (43.77)		231 (39.69)	351 (60.31)	
Energy drink	Rarely/never	3417 (72.73)	1281 (27.27)	**<0.001**	1644 (33.88)	3209 (66.12)	0.573
	Weekly	163 (59.06)	113 (40.94)		93 (32.86)	190 (67.14)	
	Daily	65 (48.15)	70 (51.85)		53 (37.86)	87 (62.14)	
Sport drink	Rarely/never	3534 (71.32)	1421 (28.68)	0.751	1752 (34.28)	3359 (65.72)	**0.008**
	Weekly	87 (70.73)	36 (29.27)		31 (23.85)	99 (76.15)	
	Daily	12 (80.00)	3 (20.00)		9 (56.25)	7 (43.75)	

Note: Values represent number and row percentage of participants within each subgroup. *p*-values are based on Pearson’s chi-squared and Fisher’s exact tests. Bold *p*-values indicate statistical significance at the 0.05 level.

**Table 2:** Prevalence of gum bleeding when brushing teeth and mobile teeth by sociodemographic, behavioral, and dietary characteristics.

Variable	Category	No Gum Bleeding (n, %)	Has Gum Bleeding (n, %)	*p*-Value	No Mobile Teeth (n, %)	Has Mobile Teeth (n, %)	*p*-Value
Sex	Male	2053 (85.40)	351 (14.60)	**0.005**	2232 (92.04)	193 (7.96)	0.44
	Female	2363 (82.56)	499 (17.44)		2611 (91.45)	244 (8.55)	
Age group	18–34	846 (78.33)	234 (21.67)	**<0.001**	1033 (96.18)	41 (3.82)	**<0.001**
	35–64	2176 (82.86)	450 (17.14)		2398 (90.97)	238 (9.03)	
	65+	1394 (89.36)	166 (10.64)		1412 (89.94)	158 (10.06)	
Education	Primary	1881 (82.68)	394 (17.32)	0.1	2017 (88.00)	275 (12.00)	**<0.001**
	Secondary	1519 (85.15)	265 (14.85)		1671 (93.88)	109 (6.12)	
	Tertiary	1016 (84.18)	191 (15.82)		1155 (95.61)	53 (4.39)	
Employment	Unemployed	2155 (85.14)	376 (14.86)	**0.015**	2287 (90.11)	251 (9.89)	**<0.001**
	Employed	2261 (82.67)	474 (17.33)		2556 (93.22)	186 (6.78)	
Residence	Rural	1393 (82.47)	296 (17.53)	0.061	1514 (89.37)	180 (10.63)	**<0.001**
	Urban	3023 (84.51)	554 (15.49)		3329 (92.83)	257 (7.17)	
Financial status	Average	2509 (83.88)	482 (16.12)	**0.009**	2755 (91.80)	246 (8.20)	**<0.001**
	Good	1315 (85.61)	221 (14.39)		1451 (94.53)	84 (5.47)	
	Poor	509 (80.28)	125 (19.72)		538 (84.06)	102 (15.94)	
Smoking status	Never	2365 (84.46)	435 (15.54)	**0.016**	2598 (92.65)	206 (7.35)	**0.006**
	Quit	818 (80.99)	192 (19.01)		929 (91.71)	84 (8.29)	
	Smokes	1195 (84.99)	211 (15.01)		1270 (89.75)	145 (10.25)	
Alcohol	Abstinent	1319 (84.55)	241 (15.45)	0.346	1403 (89.65)	162 (10.35)	**<0.001**
	<1/month	1064 (84.18)	200 (15.82)		1169 (92.56)	94 (7.44)	
	1–3/month	905 (83.49)	179 (16.51)		1029 (94.66)	58 (5.34)	
	1–4/week	676 (81.84)	150 (18.16)		773 (92.91)	59 (7.09)	
	Daily/almost daily	414 (85.71)	69 (14.29)		426 (87.47)	61 (12.53)	
Dairy frequency	Rarely/never	436 (79.13)	115 (20.87)	**0.016**	479 (86.15)	77 (13.85)	**<0.001**
	1/week	260 (83.87)	50 (16.13)		280 (89.17)	34 (10.83)	
	2–3/week	890 (84.44)	164 (15.56)		963 (91.28)	92 (8.72)	
	4+/week	2808 (84.48)	516 (15.52)		3096 (93.00)	233 (7.00)	
Juice frequency	Rarely/never	2909 (82.64)	611 (17.36)	**0.001**	3196 (90.49)	336 (9.51)	**<0.001**
	Weekly	1114 (87.10)	165 (12.90)		1214 (94.70)	68 (5.30)	
	Daily	339 (84.12)	64 (15.88)		374 (93.27)	27 (6.73)	
Soda frequency	Rarely/never	3003 (86.49)	469 (13.51)	**<0.001**	3185 (91.42)	299 (8.58)	0.286
	Weekly	948 (80.68)	227 (19.32)		1091 (92.85)	84 (7.15)	
	Daily	432 (75.00)	144 (25.00)		528 (91.35)	50 (8.65)	
Energy drink	Rarely/never	4067 (84.48)	747 (15.52)	**<0.001**	4425 (91.58)	407 (8.42)	**0.03**
	Weekly	226 (80.43)	55 (19.57)		266 (95.68)	12 (4.32)	
	Daily	98 (70.50)	41 (29.50)		124 (89.21)	15 (10.79)	
Sport drink	Rarely/never	4255 (83.93)	815 (16.07)	0.053	4665 (91.67)	424 (8.33)	0.32
	Weekly	111 (86.05)	18 (13.95)		122 (95.31)	6 (4.69)	
	Daily	10 (62.50)	6 (37.50)		15 (93.75)	1 (6.25)	

Note: Significance assessed by Pearson’s chi-square and Fisher’s exact tests. Percentages reflect the proportion of each outcome within variable categories. Statistically significant results are shown in bold (*p* < 0.05).

**Table 3:** Distribution of number of teeth missing and self-perceived oral health according to sociodemographic and behavioral variables (n, %). Significant differences are indicated in bold.

Variable	Category	Number of Teeth Missing					Self-Perceived Oral Health			
		No Missing (n, %)	1–5 Teeth Missing (n, %)	6–19 Teeth Missing (n, %)	20+ Teeth Missing (n, %)	*p*-Value	Good (n, %)	Average (n, %)	Poor (n, %)	*p*-Value
Sex	Male	87 (4.33)	998 (49.68)	551 (27.43)	373 (18.57)	**<0.001**	1105 (44.81)	814 (33.01)	547 (22.18)	0.141
	Female	96 (4.00)	1038 (43.27)	676 (28.18)	589 (24.55)		1331 (45.61)	1003 (34.37)	584 (20.01)	
Age group	18–34	67 (12.14)	441 (79.89)	39 (7.07)	5 (0.91)	**<0.001**	745 (68.10)	238 (21.76)	111 (10.15)	**<0.001**
	35–64	85 (3.64)	1281 (54.79)	689 (29.47)	283 (12.10)		1244 (46.44)	907 (33.86)	528 (19.71)	
	65+	31 (2.04)	314 (20.69)	499 (32.87)	674 (44.40)		447 (27.75)	672 (41.71)	492 (30.54)	
Education	Primary	45 (2.18)	715 (34.64)	646 (31.30)	658 (31.88)	**<0.001**	759 (32.81)	799 (34.54)	755 (32.64)	**<0.001**
	Secondary	63 (4.46)	741 (52.40)	387 (27.37)	223 (15.77)		924 (50.49)	630 (34.43)	276 (15.08)	
	Tertiary	75 (8.06)	580 (62.37)	194 (20.86)	81 (8.71)		753 (60.68)	388 (31.27)	100 (8.06)	
Employment	Unemployed	64 (2.88)	637 (28.63)	717 (32.22)	807 (36.27)	**<0.001**	909 (34.95)	960 (36.91)	732 (28.14)	**<0.001**
	Employed	119 (5.45)	1399 (64.09)	510 (23.36)	155 (7.10)		1527 (54.87)	857 (30.79)	399 (14.34)	
Residence	Rural	40 (2.74)	648 (44.38)	414 (28.36)	358 (24.52)	**<0.001**	684 (39.68)	563 (32.66)	477 (27.67)	**<0.001**
	Urban	143 (4.85)	1388 (47.08)	813 (27.58)	604 (20.49)		1752 (47.87)	1254 (34.26)	654 (17.87)	
Financial status	Average	88 (3.37)	1141 (43.75)	792 (30.37)	587 (22.51)	**<0.001**	1273 (41.52)	1110 (36.20)	683 (22.28)	**<0.001**
	Good	77 (6.58)	651 (55.59)	245 (20.92)	198 (16.91)		948 (60.96)	419 (26.95)	188 (12.09)	
	Poor	16 (2.90)	193 (34.96)	174 (31.52)	169 (30.62)		159 (24.42)	248 (38.10)	244 (37.48)	
Smoking status	Never	105 (4.65)	1123 (49.76)	564 (24.99)	465 (20.60)	**<0.001**	1436 (50.51)	954 (33.56)	453 (15.93)	**<0.001**
	Quit	29 (3.17)	346 (37.86)	296 (32.39)	243 (26.59)		395 (37.84)	404 (38.70)	245 (23.47)	
	Smokes	47 (3.93)	547 (45.70)	354 (29.57)	249 (20.80)		580 (40.25)	438 (30.40)	423 (29.35)	
Alcohol	Abstinent	42 (3.11)	485 (35.90)	381 (28.20)	443 (32.79)	**<0.001**	610 (38.41)	558 (35.14)	420 (26.45)	**<0.001**
	<1/month	37 (3.52)	515 (49.00)	300 (28.54)	199 (18.93)		623 (48.52)	418 (32.55)	243 (18.93)	
	1–3/month	33 (3.86)	499 (58.43)	209 (24.47)	113 (13.23)		594 (54.00)	350 (31.82)	156 (14.18)	
	1–4/week	54 (7.99)	361 (53.40)	175 (25.89)	86 (12.72)		416 (48.71)	295 (34.54)	143 (16.74)	
	Daily/almost daily	17 (3.88)	150 (34.25)	153 (34.93)	118 (26.94)		165 (32.67)	181 (35.84)	159 (31.49)	
Dairy frequency	Rarely/never	23 (4.86)	173 (36.58)	137 (28.96)	140 (29.60)	**<0.001**	224 (39.86)	176 (31.32)	162 (28.83)	**<0.001**
	1/week	9 (3.46)	122 (46.92)	72 (27.69)	57 (21.92)		126 (39.13)	105 (32.61)	91 (28.26)	
	2–3/week	38 (4.36)	395 (45.30)	265 (30.39)	174 (19.95)		479 (44.60)	384 (35.75)	211 (19.65)	
	4+/week	112 (4.03)	1334 (47.95)	747 (26.85)	589 (21.17)		1588 (46.79)	1144 (33.71)	662 (19.51)	
Juice frequency	Rarely/never	118 (3.89)	1279 (42.13)	914 (30.11)	725 (23.88)	**<0.001**	1480 (41.19)	1252 (34.85)	861 (23.96)	**<0.001**
	Weekly	49 (5.01)	548 (55.98)	218 (22.27)	164 (16.75)		713 (54.80)	408 (31.36)	180 (13.84)	
	Daily	16 (4.79)	176 (52.69)	75 (22.46)	67 (20.06)		213 (51.08)	130 (31.18)	74 (17.75)	
Soda frequency	Rarely/never	121 (4.05)	1255 (41.99)	867 (29.01)	746 (24.96)	**<0.001**	1566 (44.10)	1227 (34.55)	758 (21.35)	**<0.001**
	Weekly	41 (4.57)	505 (56.30)	234 (26.09)	117 (13.04)		611 (51.00)	379 (31.64)	208 (17.36)	
	Daily	20 (4.12)	254 (52.26)	115 (23.66)	97 (19.96)		237 (40.65)	190 (32.59)	156 (26.76)	
Energy drink	Rarely/never	168 (4.09)	1829 (44.53)	1164 (28.34)	946 (23.03)	**<0.001**	2184 (44.35)	1691 (34.34)	1049 (21.30)	**<0.001**
	Weekly	11 (6.04)	127 (69.78)	37 (20.33)	7 (3.85)		169 (59.30)	80 (28.07)	36 (12.63)	
	Daily	4 (4.12)	68 (70.10)	20 (20.62)	5 (5.15)		65 (46.76)	36 (25.90)	38 (27.34)	
Sport drink	Rarely/never	170 (3.98)	1940 (45.43)	1206 (28.24)	954 (22.34)	**<0.001**	2316 (44.68)	1763 (34.01)	1105 (21.32)	**<0.001**
	Weekly	9 (9.89)	66 (72.53)	11 (12.09)	5 (5.49)		85 (65.38)	30 (23.08)	15 (11.54)	
	Daily	1 (10.00)	5 (50.00)	4 (40.00)	0 (0.00)		10 (62.50)	5 (31.25)	1 (6.25)	

Note: Number of teeth missing categories: 0, 1–5, 6–19, 20+ missing teeth. Self-perceived oral health: Good, Average, Poor. Some categories may not sum exactly to 100% due to rounding.

**Table 4:** Adjusted associations between sociodemographic, behavioral, and dietary factors and oral health outcomes (having filled teeth, gum bleeding, and mobile teeth), reported as odds ratios (ORs) with 95% confidence intervals.

Variable	Category	Has Active Caries		Has Filled Teeth Due to Decay		Has Gum Bleeding	
		OR [95% CI]	*p*-Value	OR [95% CI]	*p*-Value	OR [95% CI]	*p*-Value
Sex	Male	Ref	—	Ref	—	Ref	—
	Female	**0.83 [0.72–0.96]**	**0.014**	**1.34 [1.16–1.56]**	**<0.001**	**1.55 [1.29–1.85]**	**<0.001**
Age group	18–34	Ref	—	Ref	—	Ref	—
	35–64	0.87 [0.73–1.04]	0.13	1.10 [0.91–1.34]	0.329	**0.74 [0.60–0.91]**	**0.004**
	65+	**0.36 [0.29–0.46]**	**<0.001**	**0.40 [0.32–0.50]**	**<0.001**	**0.37 [0.28–0.49]**	**<0.001**
Education	Primary	Ref	—	Ref	—	Ref	—
	Secondary	**0.60 [0.52–0.71]**	**<0.001**	**2.04 [1.75–2.38]**	**<0.001**	**0.78 [0.65–0.95]**	**0.013**
	Tertiary	**0.56 [0.46–0.69]**	**<0.001**	**3.45 [2.81–4.22]**	**<0.001**	0.90 [0.71–1.13]	0.345
Employment	Unemployed	Ref	—	Ref	—	Ref	—
	Employed	1.01 [0.86–1.20]	0.872	**2.06 [1.74–2.45]**	**<0.001**	0.95 [0.78–1.15]	0.581
Residence	Rural	Ref	—	Ref	—	Ref	—
	Urban	**0.82 [0.71–0.94]**	**0.005**	**1.17 [1.02–1.35]**	**0.029**	0.97 [0.82–1.15]	0.717
Financial status	Average	Ref	—	Ref	—	Ref	—
	Good	**0.66 [0.56–0.77]**	**<0.001**	0.95 [0.81–1.10]	0.488	**0.83 [0.69–0.99]**	**0.041**
	Poor	1.13 [0.93–1.38]	0.223	1.05 [0.86–1.28]	0.641	1.23 [0.97–1.55]	0.086
Smoking status	Never	Ref	—	Ref	—	Ref	—
	Quit	1.11 [0.92–1.33]	0.264	0.84 [0.71–1.00]	0.054	**1.41 [1.15–1.72]**	**0.001**
	Smokes	**1.42 [1.21–1.66]**	**<0.001**	**0.62 [0.53–0.73]**	**<0.001**	**0.68 [0.56–0.83]**	**<0.001**
Alcohol	Abstinent	Ref	—	Ref	—	Ref	—
	<1/month	**0.81 [0.68–0.98]**	**0.028**	1.09 [0.92–1.30]	0.332	1.01 [0.81–1.25]	0.946
	1–3/month	0.97 [0.80–1.18]	0.755	**1.31 [1.08–1.59]**	**0.007**	1.16 [0.92–1.47]	0.207
	1–4/week	0.81 [0.65–1.01]	0.064	**1.44 [1.16–1.79]**	**0.001**	**1.38 [1.07–1.77]**	**0.014**
	Daily/almost daily	1.00 [0.77–1.30]	0.997	1.20 [0.93–1.54]	0.158	1.22 [0.88–1.69]	0.222
Dairy frequency	Rarely/never	Ref	—	Ref	—	Ref	—
	1/week	1.18 [0.86–1.62]	0.304	**1.65 [1.20–2.28]**	**0.002**	0.77 [0.53–1.13]	0.187
	2–3/week	1.02 [0.80–1.30]	0.863	**1.38 [1.09–1.75]**	**0.008**	**0.71 [0.54–0.94]**	**0.017**
	4+/week	0.94 [0.75–1.16]	0.545	**1.59 [1.29–1.96]**	**<0.001**	**0.76 [0.59–0.96]**	**0.023**
Juice frequency	Rarely/never	Ref	—	Ref	—	Ref	—
	Weekly	**0.84 [0.72–0.99]**	**0.042**	0.92 [0.79–1.09]	0.338	**0.62 [0.51–0.76]**	**<0.001**
	Daily	0.85 [0.65–1.10]	0.21	0.96 [0.75–1.23]	0.737	0.88 [0.65–1.19]	0.41
Soda frequency	Rarely/never	Ref	—	Ref	—	Ref	—
	Weekly	**1.33 [1.12–1.57]**	**0.001**	1.14 [0.96–1.36]	0.143	**1.57 [1.29–1.92]**	**<0.001**
	Daily	**1.57 [1.27–1.94]**	**<0.001**	0.85 [0.69–1.06]	0.145	**1.94 [1.53–2.47]**	**<0.001**
Energy drink	Rarely/never	Ref	—	Ref	—	Ref	—
	Weekly	1.16 [0.87–1.54]	0.309	0.90 [0.66–1.22]	0.486	1.04 [0.73–1.46]	0.834
	Daily	**1.55 [1.03–2.31]**	**0.034**	0.93 [0.61–1.41]	0.734	**1.56 [1.01–2.42]**	**0.047**
Sport drink	Rarely/never	Ref	—	Ref	—	Ref	—
	Weekly	0.89 [0.58–1.37]	0.596	1.06 [0.67–1.69]	0.796	0.81 [0.48–1.37]	0.43
	Daily	**0.24 [0.06–0.94]**	**0.04**	0.67 [0.21–2.10]	0.489	1.80 [0.58–5.55]	0.305

Note: Abbreviations: OR = odds ratio; CI = confidence interval; Ref = reference category. Models adjusted for all listed covariates. Statistically significant associations are shown in bold.

**Table 5:** Multivariable associations with tooth mobility, number of missing teeth, and self-perceived oral health.

Variable	Category	Tooth Mobility		Number of Missing Teeth		Self-Perceived Oral Health	
		OR [95% CI]	*p*-Value	OR [95% CI]	*p*-Value	OR [95% CI]	*p*-Value
Sex	Male	Ref	—	Ref	—	Ref	—
	Female	1.15 [0.91–1.47]	0.242	1.14 [0.99–1.31]	0.071	0.90 [0.79–1.01]	0.08
Age group	18–34	Ref	—	Ref	—	Ref	—
	35–64	**2.13 [1.46–3.09]**	**<0.001**	**6.14 [4.85–7.77]**	**<0.001**	**2.45 [2.07–2.91]**	**<0.001**
	65+	**1.90 [1.24–2.90]**	**0.003**	**21.99 [16.67–29.00]**	**<0.001**	**3.90 [3.19–4.78]**	**<0.001**
Education	Primary	Ref	—	Ref	—	Ref	—
	Secondary	**0.66 [0.51–0.85]**	**0.001**	**0.52 [0.45–0.60]**	**<0.001**	**0.61 [0.54–0.70]**	**<0.001**
	Tertiary	**0.55 [0.39–0.77]**	**0.001**	**0.30 [0.25–0.36]**	**<0.001**	**0.47 [0.40–0.55]**	**<0.001**
Employment	Unemployed	Ref	—	Ref	—	Ref	—
	Employed	0.80 [0.61–1.05]	0.109	**0.49 [0.42–0.58]**	**<0.001**	**0.66 [0.57–0.76]**	**<0.001**
Residence	Rural	Ref	—	Ref	—	Ref	—
	Urban	**0.79 [0.64–0.98]**	**0.034**	**0.79 [0.69–0.90]**	**0.001**	**0.75 [0.67–0.85]**	**<0.001**
Financial status	Average	Ref	—	Ref	—	Ref	—
	Good	0.84 [0.64–1.10]	0.198	**0.86 [0.74–0.99]**	**0.044**	**0.60 [0.52–0.68]**	**<0.001**
	Poor	**1.77 [1.36–2.31]**	**<0.001**	1.16 [0.96–1.39]	0.13	**1.78 [1.51–2.11]**	**<0.001**
Smoking status	Never	Ref	—	Ref	—	Ref	—
	Quit	0.98 [0.74–1.30]	0.876	**1.56 [1.33–1.83]**	**<0.001**	**1.37 [1.19–1.59]**	**<0.001**
	Smokes	1.18 [0.92–1.52]	0.192	**1.81 [1.55–2.10]**	**<0.001**	**1.76 [1.53–2.02]**	**<0.001**
Alcohol	Abstinent	Ref	—	Ref	—	Ref	—
	<1/month	0.90 [0.68–1.19]	0.447	0.86 [0.73–1.01]	0.074	0.87 [0.75–1.01]	0.072
	1–3/month	0.76 [0.54–1.06]	0.108	**0.70 [0.58–0.84]**	**<0.001**	**0.81 [0.69–0.96]**	**0.013**
	1–4/week	1.00 [0.71–1.41]	0.992	**0.61 [0.50–0.75]**	**<0.001**	0.92 [0.77–1.10]	0.348
	Daily/almost daily	**1.47 [1.02–2.11]**	**0.039**	0.90 [0.71–1.14]	0.375	**1.24 [1.00–1.53]**	**0.047**
Dairy frequency	Rarely/never	Ref	—	Ref	—	Ref	—
	1/week	0.78 [0.50–1.23]	0.293	**0.63 [0.47–0.86]**	**0.004**	0.95 [0.72–1.25]	0.715
	2–3/week	0.75 [0.53–1.05]	0.092	**0.79 [0.63–0.99]**	**0.041**	0.84 [0.68–1.03]	0.092
	4+/week	**0.63 [0.47–0.85]**	**0.002**	**0.78 [0.63–0.95]**	**0.013**	0.86 [0.72–1.04]	0.115
Juice frequency	Rarely/never	Ref	—	Ref	—	Ref	—
	Weekly	**0.71 [0.53–0.94]**	**0.017**	**0.83 [0.71–0.96]**	**0.015**	**0.76 [0.66–0.86]**	**<0.001**
	Daily	0.85 [0.55–1.31]	0.455	0.94 [0.74–1.19]	0.59	**0.78 [0.63–0.96]**	**0.021**
Soda frequency	Rarely/never	Ref	—	Ref	—	Ref	—
	Weekly	0.95 [0.72–1.27]	0.742	0.97 [0.82–1.14]	0.678	1.02 [0.88–1.18]	0.76
	Daily	0.95 [0.67–1.35]	0.788	1.11 [0.90–1.37]	0.32	**1.32 [1.10–1.59]**	**0.004**
Energy drink	Rarely/never	Ref	—	Ref	—	Ref	—
	Weekly	0.65 [0.34–1.25]	0.195	0.83 [0.59–1.16]	0.27	0.80 [0.61–1.06]	0.122
	Daily	1.64 [0.86–3.12]	0.131	0.79 [0.49–1.25]	0.312	1.34 [0.91–1.96]	0.134
Sport drink	Rarely/never	Ref	—	Ref	—	Ref	—
	Weekly	1.16 [0.49–2.75]	0.735	0.93 [0.58–1.46]	0.74	1.01 [0.69–1.50]	0.944
	Daily	0.74 [0.09–6.21]	0.783	0.89 [0.24–3.35]	0.863	0.41 [0.14–1.21]	0.107

Note: Values are adjusted odds ratios (ORs) with 95% confidence intervals (CI) and *p*-values from multivariable logistic (tooth mobility) and ordinal logistic regression models (number of missing teeth; self-perceived oral health). Statistically significant associations (*p* < 0.05) are bolded. Reference categories are indicated as “Ref”.

## Data Availability

The data analyzed in this study are subject to the following licenses/restrictions: The data presented in this study are available upon request from Hungarian Central Statistical Office, which performed and supervised the data collection. Requests to access these datasets should be directed to the Hungarian Central Statistical Office, www.ksh.hu/?lang=en (accessed on 14 July 2025).

## References

[1] Oral Health. https://www.who.int/news-room/fact-sheets/detail/oral-health.

[2] Global Oral Health Status Report: Towards Universal Health Coverage for Oral Health by 2030. https://www.who.int/publications/i/item/9789240061484.

[3] Oral Health Profiles. https://www.who.int/teams/noncommunicable-diseases/surveillance/data/oral-health-profiles.

[4] Bernabe E., Marcenes W., Abdulkader R.S., Abreu L.G., Afzal S., Alhalaiqa F.N., Al-Maweri S., Alsharif U., Anyasodor A.E., Arora A. (2025). Trends in the Global, Regional, and National Burden of Oral Conditions from 1990 to 2021: A Systematic Analysis for the Global Burden of Disease Study 2021. Lancet.

[5] Frequency of Drinking Sugar-Sweetened Soft Drinks by Sex, Age and Educational Attainment Level. https://ec.europa.eu/eurostat/databrowser/view/hlth_ehis_fv7e/default/table?lang=en.

[6] (2025). Soda Consumption by Country. https://worldpopulationreview.com/country-rankings/soda-consumption-by-country.

[7] 14.1.1.27. Az Egy Főre Jutó Éves Élelmiszer-Fogyasztás Mennyisége Jövedelmi Tizedek (Decilisek) Szerint. https://www.ksh.hu/stadat_files/jov/hu/jov0026.html.

[8] (2025). Alcohol Consumption by Country. https://worldpopulationreview.com/country-rankings/alcohol-consumption-by-country.

[9] (2023). WHO Report on the Global Tobacco Epidemic. https://www.who.int/teams/health-promotion/tobacco-control/global-tobacco-report-2023.

[10] Zoltán T. 64 Millió Liter Energiaital Fogy egy Évben, Vissza Lehet Tartani Tőle a Fiatalokat?—G7—Gazdasági Sztorik Érthetően 2023. https://g7.hu/kozelet/20231004/64-millio-liter-energiaital-fogy-egy-evben-vissza-lehet-tartani-tole-a-fiatalokat.

[11] Kérdőív (2019). Európai Lakossági Egészségfelmérés. https://www.ksh.hu/elef/elef2019_kerdoiv.pdf.

[12] Ghanem A.S., Faludi E.V., Bata R., Mezei E., Hadar V., Móré M., Tóth Á., Nagy A.C. (2025). Cancer Prevalence and Its Determinants in Hungary: Analyzing Data from the 2009, 2014, and 2019 European Health Interview Surveys. PLoS ONE.

[13] Ghanem A.S., Németh O., Móré M., Nagy A.C. (2024). Role of Oral Health in Heart and Vascular Health: A Population-Based Study. PLoS ONE.

[14] Hintzpeter B., Finger J.D., Allen J., Kuhnert R., Seeling S., Thelen J., Lange C. (2019). European Health Interview Survey (EHIS) 2—Background and Study Methodology. J. Health Monit..

[15] Ulambayar B., Ghanem A.S., Nagy A.C. (2025). Overnutrition in the Elderly Population: Socio-Demographic and Behavioral Risk Factors in Hungary. Nutrients.

[16] Ulambayar B., Ghanem A.S., Tóth Á., Nagy A.C. (2025). Impact of Physical Activity and Dietary Habits on Mental Well-Being in Patients with Diabetes Mellitus. Nutrients.

[17] (2023). Stata Statistical Software, version 18.0.

[18] Lipsky M.S., Su S., Crespo C.J., Hung M. (2021). Men and Oral Health: A Review of Sex and Gender Differences. Am. J. Men’s Health.

[19] Bhardwaj A., Bhardwaj S.V. (2012). Effect of Menopause on Women’s Periodontium. J. Mid-Life Health.

[20] Boyapati R., Cherukuri S.A., Bodduru R., Kiranmaye A. (2021). Influence of Female Sex Hormones in Different Stages of Women on Periodontium. J. Mid-Life Health.

[21] Nascimento G.G., Alves-Costa S., Romandini M. (2024). Burden of Severe Periodontitis and Edentulism in 2021, with Projections up to 2050: The Global Burden of Disease 2021 Study. J. Periodontal Res..

[22] Gupta S., Mukhiya S., Kafle A., Ghimire S., Acharya U.K. (2023). Tooth Mobility among Patients Visiting a Tertiary Care Centre: A Descriptive Cross-Sectional Study. J. Nepal Med. Assoc..

[23] Borges K., Faria C.S., Silva C.M., Mendonça L. (2022). Self-perceived oral health among the elderly in four family health units. Aten. Primaria.

[24] Slade G.D., Sanders A.E. (2011). The Paradox of Better Subjective Oral Health in Older Age. J. Dent. Res..

[25] Pengpid S., Peltzer K. (2023). Poor Self-Rated Oral Health Status and Associated Factors Amongst Adults in Algeria. Int. Dent. J..

[26] Gomes A.P., da Silva E.G., Gonçalves S.H.F., Huhtala M.F.R.L., Martinho F.C., Gonçalves S.E.P. (2015). Relationship between Patient’s Education Level and Knowledge on Oral Health Preventive Measures. Int. Dent. Med. J. Adv. Res..

[27] de Andrade F.B., Antunes J.L.F. (2025). Evaluating Socioeconomic Inequalities in Self-Rated Oral Health and Its Contributing Factors in Brazilian Older Adults. PLoS ONE.

[28] Al-Sudani F.Y.H., Vehkalahti M.M., Suominen A.L. (2015). The Association between Current Unemployment and Clinically Determined Poor Oral Health. Community Dent. Oral Epidemiol..

[29] Hakeberg M., Boman U.W. (2017). Self-Reported Oral and General Health in Relation to Socioeconomic Position. BMC Public Health.

[30] Luo H., Wu Q., Bell R.A., Wright W., Quandt S.A., Basu R., Moss M.E. (2021). Rural-Urban Differences in Dental Service Utilization and Dental Service Procedures Received among US Adults: Results from the 2016 Medical Expenditure Panel Survey. J. Rural Health.

[31] Costa M.J.F., Lins C.A.d.A., de Macedo L.P.V., de Sousa V.P.S., Duque J.A., de Souza M.C. (2019). Clinical and Self-Perceived Oral Health Assessment of Elderly Residents in Urban, Rural, and Institutionalized Communities. Clinics.

[32] Thompson B., Cooney P., Lawrence H., Ravaghi V., Quiñonez C. (2014). Cost as a Barrier to Accessing Dental Care: Findings from a Canadian Population-Based Study. J. Public Health Dent..

[33] Probst L.F., Junior G.A.P., Pereira A.C., De Carli A.D. (2019). Impact of financial crises on oral health indicators: An integrative review of the literature. Cienc. Saude Coletiva.

[34] Cianetti S., Valenti C., Orso M., Lomurno G., Nardone M., Lomurno A.P., Pagano S., Lombardo G. (2021). Systematic Review of the Literature on Dental Caries and Periodontal Disease in Socio-Economically Disadvantaged Individuals. Int. J. Environ. Res. Public Health.

[35] Molarius A., Engström S., Flink H., Simonsson B., Tegelberg Å. (2014). Socioeconomic Differences in Self-Rated Oral Health and Dental Care Utilisation after the Dental Care Reform in 2008 in Sweden. BMC Oral Health.

[36] Hagh L.G., Zakavi F., Ansarifar S., Ghasemzadeh O., Solgi G. (2013). Association of Dental Caries and Salivary sIgA with Tobacco Smoking. Aust. Dent. J..

[37] Jia Y.-J., Liao Y., He Y.-Q., Zheng M.-Q., Tong X.-T., Xue W.-Q., Zhang J.-B., Yuan L.-L., Zhang W.-L., Jia W.-H. (2021). Association Between Oral Microbiota and Cigarette Smoking in the Chinese Population. Front. Cell. Infect. Microbiol..

[38] Jiang X., Jiang X., Wang Y., Huang R. (2019). Correlation between Tobacco Smoking and Dental Caries: A Systematic Review and Meta-Analysis. Tob. Induc. Dis..

[39] Csikar J., Kang J., Wyborn C., Dyer T.A., Marshman Z., Godson J. (2016). The Self-Reported Oral Health Status and Dental Attendance of Smokers and Non-Smokers in England. PLoS ONE.

[40] Drilea S.K., Reid B.C., Li C.-H., Hyman J.J., Manski R.J. (2005). Dental Visits among Smoking and Nonsmoking US Adults in 2000. Am. J. Health Behav..

[41] Nair P., Sutherland G., Palmer R.M., Wilson R.F., Scott D.A. (2003). Gingival Bleeding on Probing Increases after Quitting Smoking. J. Clin. Periodontol..

[42] Zhang J., Yu J., Dou J., Hu P., Guo Q. (2021). The Impact of Smoking on Subgingival Plaque and the Development of Periodontitis: A Literature Review. Front. Oral Health.

[43] Zappacosta B., Persichilli S., De Sole P., Mordente A., Giardina B. (1999). Effect of Smoking One Cigarette on Antioxidant Metabolites in the Saliva of Healthy Smokers. Arch. Oral Biol..

[44] Mai X., Wactawski-Wende J., Hovey K.M., LaMonte M.J., Chen C., Tezal M., Genco R.J. (2013). Associations between Smoking and Tooth Loss According to the Reason for Tooth Loss. J. Am. Dent. Assoc..

[45] Wu L.-Z., Duan D.-M., Liu Y.-F., Ge X., Zhou Z.-F., Wang X.-J. (2013). Nicotine Favors Osteoclastogenesis in Human Periodontal Ligament Cells Co-Cultured with CD4+ T Cells by Upregulating IL-1β. Int. J. Mol. Med..

[46] Krall E.A., Dawson-Hughes B. (1999). Smoking Increases Bone Loss and Decreases Intestinal Calcium Absorption. J. Bone Miner. Res..

[47] Gandhi U.H., Benjamin A., Gajjar S., Hirani T., Desai K., Suhagia B.B., Ahmad R., Sinha S., Haque M., Kumar S. (2024). Alcohol and Periodontal Disease: A Narrative Review. Cureus.

[48] Wang J., Lv J., Wang W., Jiang X. (2016). Alcohol Consumption and Risk of Periodontitis: A Meta-Analysis. J. Clin. Periodontol..

[49] Colaco A.S., Mayya A., Shetty P., Mayya S.S. (2024). Dental Impact on Daily Life and Oral Health in Alcohol Use Disorder Patients. Clin. Epidemiol. Glob. Health.

[50] Morse D.E., Avlund K., Christensen L.B., Fiehn N.-E., Molbo D., Holmstrup P., Kongstad J., Mortensen E.L., Holm-Pedersen P. (2014). Smoking and Drinking as Risk Indicators for Tooth Loss in Middle-Aged Danes. J. Aging Health.

[51] Heegaard K., Avlund K., Holm-Pedersen P., Hvidtfeldt U.A., Bardow A., Grønbæk M. (2011). Amount and Type of Alcohol Consumption and Missing Teeth among Community-Dwelling Older Adults: Findings from the Copenhagen Oral Health Senior Study. J. Public Health Dent..

[52] Enberg N., Wolf J., Ainamo A., Alho H., Heinälä P., Lenander-Lumikari M. (2001). Dental Diseases and Loss of Teeth in a Group of Finnish Alcoholics: A Radiological Study. Acta Odontol. Scand..

[53] Min E.J., Park E., Park J.-B. (2024). Milk Consumption and Its Association with Dental Caries: Gender-Specific Insights from the Korea National Health and Nutrition Examination Survey (2013–2015). Medicina.

[54] Gupta P., Gupta N., Pawar A.P., Birajdar S.S., Natt A.S., Singh H.P. (2013). Role of Sugar and Sugar Substitutes in Dental Caries: A Review. Int. Sch. Res. Not..

[55] Levine R.S. (2001). Milk, Flavoured Milk Products and Caries. Br. Dent. J..

[56] Utsunomiya H., Tanaka K., Okubo H., Nagata C., Miyake Y. (2025). Association between Dairy Product Intake and Prevalence of Dental Caries in 3-Year-Old Japanese Children. J. Pediatr. Gastroenterol. Nutr..

[57] Marcone S., Belton O., Fitzgerald D.J. (2017). Milk-Derived Bioactive Peptides and Their Health Promoting Effects: A Potential Role in Atherosclerosis. Br. J. Clin. Pharmacol..

[58] Lee K., Kim J. (2019). Dairy Food Consumption Is Inversely Associated with the Prevalence of Periodontal Disease in Korean Adults. Nutrients.

[59] Vitiello F., Bourgeois D., Orilisi G., Orsini G., Carrouel F. (2024). Non-Cariogenic Effect of Milk and Dairy Products on Oral Health in Children and Adolescents: A Scoping Review. Children.

[60] Li A., Ma Y., Cui N., Zhang X., Zheng Q., Du P., Sun M. (2023). Research Progress of Milk and Dairy Products to Prevent Caries. J. Funct. Foods.

[61] Cao R., Li A., Geng F., Pan Y. (2024). Associations of Dietary Antioxidant Intake with Periodontal Health among US Adults: An Exploratory Mediation Analysis via Mitochondrial Function. J. Clin. Periodontol..

[62] Office of Dietary Supplements—Vitamin C. https://ods.od.nih.gov/factsheets/VitaminC-HealthProfessional/.

[63] Inchingolo A.M., Malcangi G., Ferrante L., Del Vecchio G., Viapiano F., Mancini A., Inchingolo F., Inchingolo A.D., Di Venere D., Dipalma G. (2023). Damage from Carbonated Soft Drinks on Enamel: A Systematic Review. Nutrients.

[64] Salim A., Angelova S., Roussev B., Sokrateva T., Galunska B., Olczyk P., Komosinska-Vassev K., Peev S., Kiselova-Kaneva Y., Ivanova D. (2025). Association Between Frequency of Sugar and Protein Intake and Severity of Plaque-Induced Gingivitis in Children. Turk. Arch. Pediatr..

[65] Gupta V., Dawar A., Bhadauria U.S., Purohit B.M., Nilima N. (2023). Sugar-Sweetened Beverages and Periodontal Disease: A Systematic Review. Oral Dis..

[66] Sim E., Sohn W., Choi E., Noh H. (2022). Oral Health Impact of Sugar-Sweetened Beverage Consumption among Korean Adolescents. Int. J. Dent. Hyg..

[67] Erdemir U., Yildiz E., Saygi G., Altay N.I., Eren M.M., Yucel T. (2016). Effects of Energy and Sports Drinks on Tooth Structures and Restorative Materials. World J. Stomatol..

[68] Manuelli M., Marcolina M., Nardi N., Bertossi D., De Santis D., Ricciardi G., Luciano U., Nocini R., Mainardi A., Lissoni A. (2019). Oral Mucosal Complications in Orthodontic Treatment. Minerva Stomatol..

[69] Hooper S., West N.X., Sharif N., Smith S., North M., De’Ath J., Parker D.M., Roedig-Penman A., Addy M. (2004). A Comparison of Enamel Erosion by a New Sports Drink Compared to Two Proprietary Products: A Controlled, Crossover Study in Situ. J. Dent..

